# Enhanced Systemic Response of Matrix Metalloproteinases and Their Regulators in *Campylobacter* and *Salmonella* Patients

**DOI:** 10.3390/diagnostics8040082

**Published:** 2018-12-13

**Authors:** Anna Nilsson, Taina Tervahartiala, David Lennebratt, Anders Lannergård, Timo Sorsa, Hilpi Rautelin

**Affiliations:** 1 Department of Medical Sciences, Clinical Microbiology, Uppsala University, Dag Hammarskjölds väg 38, 75237 Uppsala, Sweden; anna.nilsson@medsci.uu.se; 2 Department of Oral and Maxillofacial Diseases, Helsinki University Central Hospital, Stenbäckinkatu 9, PO Box 100, 00029 Helsinki, Finland; taina.tervahartiala@helsinki.fi; 3 Department of Medical Sciences, Infectious Diseases, Uppsala University, SE-75185 Uppsala, Sweden; david.lennebratt@akademiska.se (D.L.); anders.lannergard@akademiska.se (A.L.); 4 Department of Dental Medicine, Division of Periodontology, Karolinska Institute, SE-17177 Stockholm, Sweden; timo.sorsa@helsinki.fi

**Keywords:** *Campylobacter*, *Salmonella*, enteritis, matrix metalloproteinase (MMP)-8, matrix metalloproteinase (MMP)-9, tissue inhibitor of metalloproteinases (TIMP)-1, myeloperoxidase, human neutrophil elastase

## Abstract

Campylobacters are major enteropathogens worldwide with a substantial financial burden. Matrix metalloproteinases (MMPs) are proteolytic metalloendopeptidases with ability to modify immune response and shown to be upregulated in patients with several tissue destructive diseases, including infections. We measured here serum concentrations of MMP-8 and MMP-9 together with their regulators myeloperoxidase (MPO), human neutrophil elastase (HNE), and tissue inhibitor of metalloproteinases (TIMP)-1 in 80 *Campylobacter* and 25 *Salmonella* patients as well as in 27 healthy controls. Paired serum samples were available for 73 and 23 patients, respectively. When the initial serum samples were compared to those from controls, both *Campylobacter* and *Salmonella* patients showed elevated concentrations of all biomarkers tested (*p* ≤ 0.037). In the follow-up samples, collected about 25 days afterwards, MMP-8 levels of *Campylobacter* patients had already turned to normal but all the other biomarkers still showed elevated, although from the initial levels significantly dropped, levels. For the follow-up samples of *Salmonella* patients, only MMP-9 and MPO levels were at a significantly higher level than in controls. It remains to be studied if the systematically enhanced neutrophil-derived proteolytic and oxidative stress, induced by *Campylobacter* infection as shown here and persisting for several weeks, is important for the development of late sequelae.

## 1. Introduction

*Campylobacter* is the most common cause of bacterial gastroenteritis in most parts of the world and the financial burden of the infections in the European Union alone is estimated to be 2.4 billion euro every year [1,2]. The symptoms of gastroenteritis caused by *Campylobacter* can vary from mild with watery diarrhea, fever, and abdominal cramps to more severe with bloody stools and vomiting [3]. Occasionally *Campylobacter* may even cause bacteraemia, which has been shown to occur in less than 1% of the total number of *Campylobacter* infections [4,5]. Even though the outcome of *Campylobacter* infections is generally good, late sequelae such as Guillain–Barré Syndrome [6], reactive arthritis [7] and irritable bowel syndrome [8] may develop.

The matrix metalloproteinase (MMP) family consists of genetically distinct but structurally related metalloendopeptidases with the ability to degrade almost all extracellular matrix components and to modify immune responses [9]. MMP-8 and MMP-9 are mainly stored in neutrophils and released as proenzymes during the inflammatory process. The proenzymes are locally activated and involved in leucocyte recruitment to the inflammation site [10]. MMPs are regulated at different levels; transcription and secretion, activation of the proenzyme, and local activity of MMP are regulated by tissue inhibitors of metalloproteinases (TIMPs) [9,11]. Myeloperoxidase (MPO) is a cationic heme protein released upon neutrophil degranulation [9]. MPO is upregulated during inflammation and involved in the regulation of neutrophil migration to inflammation site [10]. Also, MPO can, by generating oxidants, activate MMPs and inactivate TIMPs [10,12]. Degranulation of neutrophils also releases human neutrophil elastase (HNE), which is a serine protease involved in host response to bacteria and can activate latent proMMPs and inactivate their endogenous inhibitors, TIMPs [10].

Elevated MMP levels have been detected also in patients with gastrointestinal diseases, such as Crohn´s disease and ulcerative colitis, in which increased MMP levels have been suggested to exert a pathogenic role [13,14,15]. We have earlier shown enhanced systemic MMP response in adult patients [16] but not in children [17] with *Helicobacter pylori* infection. Furthermore, in several experimental studies, Gram-negative bacteria have been shown to induce the RNA expression of MMPs (reviewed in [18]). In one such study in a mouse model, an upregulated transcription of some MMPs, such as MMP-8, was detected following experimental infection with the enteric pathogens *Salmonella* Typhimurium and *Yersinia enterocolitica* [19]. In the very same study, the expression of TIMP-1 was also upregulated in *Y. enterocolitica* infection [19].

Here, we wanted to study the levels of neutrophil-derived MMP-8 and MMP-9, as well as their regulators MPO, HNE and TIMP-1, in patients with *Campylobacter* infections. Serum samples were collected from *Campylobacter* and, for comparison, *Salmonella* positive patients together with healthy donors. To better understand the kinetics of the biomarkers, follow-up samples were also collected from the enteritis patients. Studies on circulating levels of MMPs in *Campylobacter* patients are scarce and this is, to the best of our knowledge, the first showing the serum levels of MMP-8.

## 2. Materials and Methods

### 2.1. Patients and Serum Samples

In this prospective, observational study, serum samples were collected from *Campylobacter jejuni/coli* and non-typhoidal *Salmonella enterica* positive adult patients who had visited the Department of Infectious Diseases at Uppsala University Hospital due to an acute enteritis and whose infections were verified by routine stool culture at the Clinical Microbiology Laboratory at Uppsala University Hospital in 2009–2013. Characteristics of the patients included are shown in Table 1. All the patients gave their written informed consent. In addition, serum samples were collected from 27 anonymous blood donors. The study was approved by the local ethical committee of Uppsala, Sweden (Dnro 2009/207, 22 July 2009).

### 2.2. Measurement of Serum MMPs and TIMP-1 Concentrations

All serum concentrations of MMP-9, TIMP-1, MPO, and HNE were determined using commercially available enzyme-linked immunosorbent assay (ELISA) kits as earlier described [16,17] except for MMP-8, which was analysed by immunofluorometric assay (IFMA, Medix Biochemica, Espoo, Finland) [20]. The determinations were performed in random order and blinded to the clinical status and samples were assayed according to the instructions of the manufacturers. The levels of the studied MMPs, TIMP-1, MPO, and HNE were expressed as ng per mL, and the detection limit for MMP-8 was 0.08 ng/mL and for the others as early described [16,17]. The concentration units (ng/mL) for calculation of MMP/TIMP-1 molar ratios, were converted to molarity units (mol/L) by using the molecular weights of MMP-8, MMP-9 and TIMP-1, respectively [16,17,20,21].

### 2.3. Statistical Analyses

Statistical analyses were performed using XLSTAT (Addinsoft, Paris, France). Biomarker data between the groups were compared using Mann–Whitney test and the Wilcoxon signed rank test was used to compare biomarker concentrations in paired serum samples. Distribution of gender in the two patient groups was compared using Fisher´s exact test. Differences in the age of the patients as well as in the time intervals between the onset of symptoms and the first serum sample, and between the first and the second serum sample collected, between *Campylobacter* and *Salmonella* patients, were calculated using Mann–Whitney test. P-values less than 0.05 were considered significant.

## 3. Results

Acute-phase serum samples from a total of 80 *Campylobacter* (38 females; age: 18–77 years old, median 48 years) and 25 *Salmonella* (19 females; age: 18–81 years old, median 53 years) positive subjects were collected. There was no significant difference between *Campylobacter* and *Salmonella* patients as far as the age was concerned but there were significantly more female patients among *Salmonella* patients (*p* = 0.020). The onset of the symptoms was known for 78 *Campylobacter* and for all 25 *Salmonella* patients and for them, there was no significant difference in the time interval between the start of the symptoms and the collection of the first serum sample, which was a median of 12 days (range, 4–67 days) and 14 days (range, 4–27 days), respectively.

**Figure 1:**
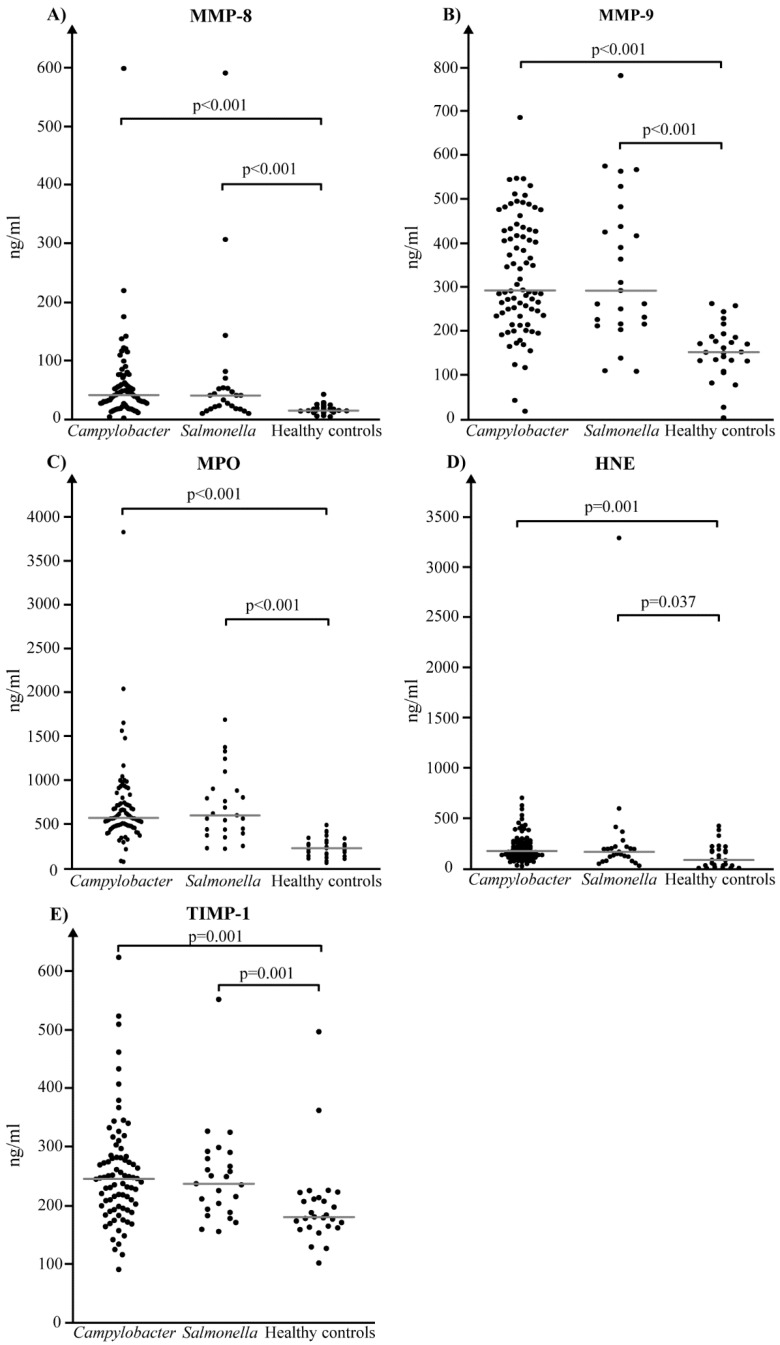
Acute-phase serum levels of matrix metalloproteinase (MMP)-8 (**A**), MMP-9 (**B**), myeloperoxidase (MPO) (**C**), human neutrophil elastase (HNE) (**D**) and tissue inhibitor of metalloproteinases (TIMP)-1 (**E**) in 80 *Campylobacter* patients, 25 *Salmonella* patients and 27 healthy donors. Data of two groups were compared using Mann–Whitney test. The grey horizontal line indicates the median.

Acute-phase serum concentrations of MMP-8, MMP-9, MPO, HNE and TIMP-1 (Table 2) were significantly higher in both *Campylobacter* and *Salmonella* patients as compared to the healthy controls (HNE, *Salmonella* patients, p=0.037; all other p-values *p* ≤ 0.001) (Figure 1). However, when these values were compared between *Campylobacter* and *Salmonella* patients, no significant differences were detected in any of the biomarkers tested. Paired serum samples were available for 73 of 80 *Campylobacter* and for 23 of 25 *Salmonella* patients. The convalescent serum samples were collected 25 days (median; range, 17–54 days) and 24 days (median; range 15–35 days) after the first samples from *Campylobacter* and *Salmonella* patients, respectively. No significant differences in the timing of the collected samples between the two patient groups were detected.

**Figure 2:**
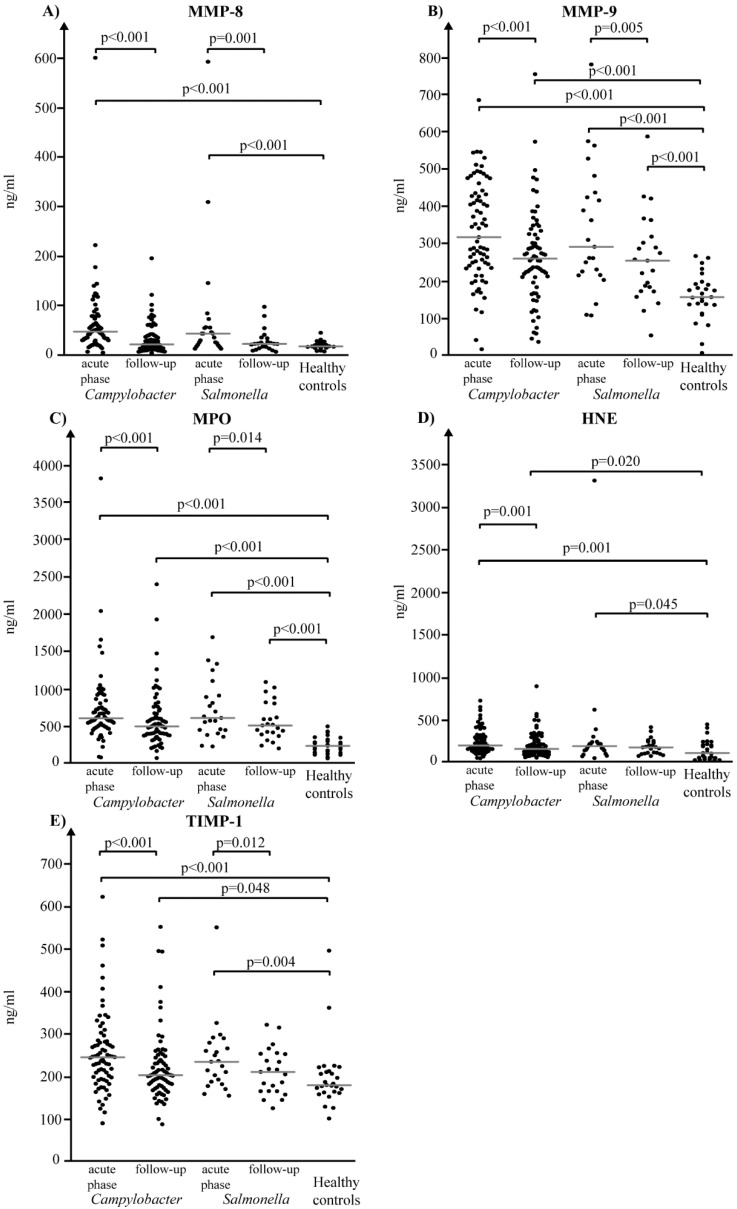
Serum levels of matrix metalloproteinase (MMP)-8 (**A**), MMP-9 (**B**), myeloperoxidase (MPO) (**C**), human neutrophil elastase (HNE) (**D**) and tissue inhibitor of metalloproteinases (TIMP)-1 (**E**) in paired samples from 73 *Campylobacter* patients, 23 *Salmonella* patients, and 27 healthy donors. The paired samples were compared using Wilcoxan signed-rank test and comparison to the healthy control group was performed using the Mann–Whitney test. The grey horizontal line indicates median.

When the acute-phase and convalescent serum samples from *Campylobacter* patients were compared to each other, significantly lower levels were demonstrated in the follow-up serum samples for all the biomarkers tested (all *p*-values ≤ 0.001) (Figure 2). A significant drop in the follow-up samples as compared to the acute-phase samples was also demonstrated in *Salmonella* patients for all biomarkers except for HNE (MMP-8, *p* = 0.001; MMP-9, *p* = 0.005; TIMP-1, *p* = 0.012; MPO, *p* = 0.014) (Figure 2). As the MMP-8/TIMP-1 and MMP-9/TIMP-1 ratios were concerned in enteritis patients, significant differences between acute and convalescent samples were detected except for MMP-9/TIMP-1 ratio for *Salmonella* patients.

When the follow-up samples of *Campylobacter* patients were compared to samples collected from healthy controls, serum concentrations of MMP-8 had already returned to levels non-significantly different from those measured in controls but the levels of all other biomarkers tested were still significantly higher than those detected in controls (MMP-9, *p* < 0. 001; TIMP-1, *p* = 0.048; MPO, *p* < 0.001; HNE, *p* = 0.02) (Figure 2). For *Salmonella* patients, serum concentrations of MMP-8, TIMP-1 and HNE had returned to normal levels in the follow-up samples whereas MMP-9 and MPO levels were still significantly higher than those in controls (both *p*-values < 0.001) (Figure 2). For both *Campylobacter* and *Salmonella* patients, MMP-9/TIMP-1 ratios were significantly higher as compared to the controls (for both, *p* < 0.001).

A total of 22 *Campylobacter* patients and nine *Salmonella* patients were hospitalized due to their infections. The median time at hospital was three days (range, 1–21 days) for *Campylobacter* patients and three days (range, 2–30 days) for *Salmonella* patients, respectively. Paired serum samples were available for 20 *Campylobacter* and eight *Salmonella* patients treated at hospital. We wanted to see if a more severe infection, defined here as hospitalization, could be reflected in the biomarker levels. Among *Campylobacter* patients, there were no significant differences in any of the biomarker levels tested, when acute-phase and follow-up samples of hospitalized patients were compared to those of outpatients. However, *Campylobacter* patients not treated at hospital had higher initial MMP-8 levels (p<0.001) and higher MMP-9 levels in follow-up (*p* < 0.001) as compared to the corresponding samples from *Salmonella* patients. For *Salmonella* patients, serum concentrations of MMP-8 and TIMP-1 were significantly higher in the acute-phase samples of hospitalized patients compared to those of outpatients (for both, *p* = 0.023).

A total of 21 *Campylobacter* patients were treated with antimicrobials (nine hospitalized and 12 outpatients) but did not show significantly higher initial levels of any biomarkers tested as compared to those who did not receive antimicrobial therapy. However, those treated had significantly lower levels of MMP-8 in their follow-up samples as compared to the follow-up samples of patients not treated with antimicrobials (*p* = 0.041). For *Salmonella* patients, ten patients, eight of which were hospitalized, received antimicrobial therapy and showed no significant differences in any of the biomarker levels tested as compared to the *Salmonella* patients not treated with antimicrobials. No *Campylobacter or Salmonella* patients were treated with tetracyclines.

## 4. Discussion

In the present study, we wished to analyse the serum concentrations of MMP-8 and MMP-9 related to the key regulators MPO, HNE and TIMP-1 in *Campylobacter* patients. In order to study the kinetics of these proinflammatory biomarkers in acute infection, both acute-phase and follow-up serum samples were included. In the initial serum samples as compared to samples from healthy controls, both *Campylobacter* and *Salmonella* patients showed elevated concentrations of all biomarkers tested. In the follow-up serum samples of *Campylobacter* patients, all biomarkers except MMP-8, still showed elevated, although from the initial levels significantly dropped, levels. For *Salmonella* patients, three of the five initially elevated biomarkers had declined to normal levels in the follow-up samples.

Elevated MMP levels have been detected in patients with gastrointestinal diseases, such as Crohn´s disease and ulcerative colitis [13,14] and *H. pylori* infection [16]. As *Campylobacter* infections are concerned, the data are scarce but some experimental studies have suggested MMPs, such as MMP-9, to have a role in campylobacteriosis in mice [22,23]. In a study on childhood gastroenteritis, two *Campylobacter* and four *Salmonella* patients were included and showed elevated serum concentrations of MMP-9 and TIMP-1 as compared to healthy controls [24]. Here, we could show enhanced systemic response of MMP-8 and MMP-9 as well as of their regulators MPO, HNE and TIMP-1 in both *Campylobacter* and *Salmonella* patients. In this regard, the selected and analyzed biomarkers (MPO, HNE, MMP-8, MMP-9, and TIMP-1) form a/an pro/oxidative and –proteolytic tissue destructive activation cascade. MPO can oxidatively activate MMP-8 and MMP-9 as well as inactivate TIMP-1, and HNE can proteolytically activate MMP-9 and inactivate TIMP-1. In concert they accelerate and promote inflammation-induced tissue degeneration [9,10]. To the best of our knowledge this is the first report to demonstrate elevated circulating MMP-8 levels in enteritis patients.

In order to study the kinetics of the biomarkers, follow-up serum samples were analysed. In *Campylobacter* patients, MMP-8 showed the most rapid changes as the initially elevated serum levels had already turned to normal during follow-up, as compared to the samples of blood donors. For the other biomarkers tested, although the initial levels detected in the first samples had significantly declined during follow-up, the serum concentrations were still significantly higher than those measured in blood donors. In *Salmonella* patients, as compared to blood donors, only MMP-9 and MPO levels remained significantly elevated after follow-up. Longstanding elevation of neutrophil degranulation products in serum after acute enteritis reflects accelerated proteolysis and oxidative stress, which in turn may contribute to intestinal and extraintestinal sequelae. In a recent study on experimental chicken model, enhanced production of MMP-9 correlated with pathology in *C. jejuni-*induced Guillain-Barré syndrome [25]. Furthermore, in much older reports, MMPs, MMP-8 in particular, have been shown to be important in reactive arthritis [26], a frequent sequela of bacterial enteritis including that caused by *Campylobacter* [7].

Although *Campylobacter* infection in most of the patients is a self-limited disease, hospitalization and/or antimicrobial therapy might be needed in severe infections. As 28% of *Campylobacter* patients in the present study needed hospital treatment, we wanted to see whether these particular individuals, with presumably a more severe infection, would show higher serum concentrations of the biomarkers tested on the baseline. In contrast to *Salmonella* patients, there were no significant differences in any of the biomarker levels between inpatients and outpatients. However, interestingly, as the biomarker levels were compared between *Campylobacter* and *Salmonella* patients, *Campylobacter* patients not treated at hospital had initially higher MMP-8 levels than *Salmonella* outpatients. This finding remains to be confirmed and it should be kept in mind that the number of *Salmonella* patients was quite limited in the present study. 

*Campylobacter* infections are not often treated with antimicrobials in Sweden, which was also reflected in the present study as only one fifth of the outpatients and less than half of the patients treated at hospital received antimicrobial therapy. *Campylobacter* patients treated with antimicrobials, as compared to those who did not receive therapy, did not show higher initial levels in any of the biomarkers tested but MMP-8 levels were significantly lower in the follow-up samples. Tetracyclines are well-known directly to inhibit MMPs [9] but no patient in this study was treated with this group of antimicrobials. The rapid decline in MMP-8 levels may reflect a lower bacterial load after successful therapy and fewer triggers for MMP production.

Although high biomarker levels could be demonstrated in the first serum samples collected, these initial samples could be collected only after the diagnosis was confirmed with culture, a median of 12 to 14 days after the onset of symptoms. It is tempting to speculate that earlier samples could have revealed even higher MMP levels. On the other hand, the follow-up was quite long as it in general took more than three weeks before the second serum sample was collected after the initial sample. Because of the long follow-up, it was possible to see that so many biomarkers, both in *Campylobacter* and *Salmonella* patients, remained elevated many weeks after the onset of the disease.

In conclusion, after human *Campylobacter* enteritis, the serum levels of MMP-8, MMP-9 and their regulators MPO, HNE and TIMP-1 are elevated as compared to the serum levels of healthy controls. This systematically enhanced neutrophil-derived proteolytic and oxidative stress seems to persist for several weeks. It remains to be studied if this increased production of MMPs and their regulators plays a role in the development of late sequelae after *Campylobacter* infection. 

## Figures and Tables

**Table 1:** Characteristics of *Campylobacter* and *Salmonella* patients included.

Characteristic	*Campylobacter* Patients	*Salmonella* Patients
Total number of patients (n)	80	25
Gender (Males/Females)	42/38	6/19
Age, median (range), years	48 (18–77)	53 (18–81)
* Treated with antimicrobials (n)	21	10
Hospitalized (n)	22 ^a^	9 ^b^

* None of the patients was treated with tetracyclines. Of the hospitalized patients, 9 *Campylobacter* ^a^ and 8 *Salmonella* ^b^ patients received antimicrobial therapy.

**Table 2:** Serum concentrations of matrix metalloproteinase (MMP)-8, MMP-9, myeloperoxidase (MPO), human neutrophil elastaste (HNE) and tissue inhibitor of metalloproteinases (TIMP)-1 in 80 *Campylobacter* and 25 *Salmonella* patients and 27 healthy controls. Paired samples were available for 73 *Campylobacter* and 23 *Salmonella* patients. Values are shown as median (25–75% percentiles).

Group		MMP-8 (IQR), (ng/mL)	MMP-9 (IQR), (ng/mL)	MPO (IQR), (ng/mL)	HNE (IQR), (ng/mL)	TIMP-1 (IQR), (ng/mL)
*Campylobacter*	acute phase n = 80	43.2 (28.7–61.5)	291.5 (234.1–427.9)	570.0 (480.0–736.4)	171.6 (112.2–265.7)	245.2 (197.5–282.3)
	follow-up n = 73	20.8 (12.0–37.6)	259.6 (209.7–323.9)	487.7 (370.4–656.8)	130.7 (93.6-232.6)	203.7 (178.2–245.0)
*Salmonella*	acute phase n = 25	42.4 (19.6–54.4)	291.2 (215.4–436.6)	600.1 (437.5–881.8)	163.0 (113.3–212.1)	236.9 (193.4–279.7)
	follow-up n = 23	21.6 (14.2–29.2)	254.3 (177.8–310.1)	500.0 (391.6–700.5)	148.5 (81.8–209.7)	211.3 (166.1–253.4)
Control	- n = 27	16.7 (14.8–22.5)	151.4 (131.2–185.6)	224.7 (145.7–299.5)	82.0 (26.3–181.0)	180.4 (163.9–212.0)

IQR: Interquartile range from 25th percentile to 75th percentile.
